# Age‐ and duration‐dependent effects of whey protein on high‐fat diet‐induced changes in body weight, lipid metabolism, and gut microbiota in mice

**DOI:** 10.14814/phy2.14523

**Published:** 2020-08-03

**Authors:** Serena Boscaini, Raul Cabrera‐Rubio, Oleksandr Nychyk, John Roger Speakman, John Francis Cryan, Paul David Cotter, Kanishka N. Nilaweera

**Affiliations:** ^1^ Food Biosciences Department Teagasc Food Research Centre, Moorepark Fermoy Ireland; ^2^ APC Microbiome Ireland University College Cork Cork Ireland; ^3^ Department of Anatomy and Neuroscience University College Cork Cork Ireland; ^4^ State Key Laboratory of Molecular Developmental Biology Institute of Genetics and Developmental Biology Chinese Academy of Sciences Beijing China; ^5^ Institute of Biological and Environmental Sciences University of Aberdeen Aberdeen Scotland

**Keywords:** energy balance, gut microbiota, high‐fat diet, lipids catabolism, nutrient transporters expression, shotgun, whey protein

## Abstract

Bovine whey protein has been demonstrated to exert a positive effect on energy balance, lipid metabolism, and nutrient absorption. Additionally, it affects gut microbiota configuration. Thus, whey protein is considered as good dietary candidate to prevent or ameliorate metabolic diseases, such as obesity. However, the relationship that links energy balance, metabolism, and intestinal microbial population mediated by whey protein intake remains poorly understood. In this study, we investigated the beneficial effects attributed to whey protein in the context of high‐fat diet (HFD) in mice at two different ages, with short or longer durations of whey protein supplementation. Here, a 5‐week dietary intervention with HFD in combination with either whey protein isolate (WPI) or the control nonwhey milk protein casein (CAS) was performed using 5‐week or 10‐week‐old C57BL/6J mice. Notably, the younger mice had no prior history of ingestion of WPI, while older mice did. 5‐week‐old HFD‐WPI‐fed mice showed a decrease in weight gain and changes in the expression of genes within the epidydimal white adipose tissue including those encoding leptin, inflammatory marker CD68, fasting‐induced adipose factor FIAF and enzymes involved in fatty acids catabolism, relative to HFD‐CAS‐fed mice. Differences in β‐diversity and higher proportions of *Lactobacillus murinus*, and related functions, were evident within the gut microbiota of HFD‐WPI mice. However, none of these changes were observed in mice that started the HFD dietary intervention at 10‐weeks‐old, with an extended period of WPI supplementation. These results suggest that the effect of whey protein on mouse body weight, adipose tissue, and intestinal parameters depends on diet duration and stage of life during which the diet is provided. In some instances, WPI influences gut microbiota composition and functional potential, which might orchestrate observed metabolic and physiological modifications.

## 1 INTRODUCTION

Bovine whey protein is a milk protein present in the liquid that results from cheese production, called whey. Whey protein includes subcategories of proteins present in different quantities, such as α‐lactalbumin (~25%), β‐lactoglobulin (~65%), bovine serum albumin (~8%), lactoperoxidase (0.25%–0.5%), lactoferrin (~1%), and minor proteins such as immunogloblulins (<1%) (Morr & Ha, 1993). Because of the specific amino acid composition including branched chain amino acids, intake of these proteins has been shown to bring about several health benefits. Notably, a diet supplemented with whey protein has been found to modulate appetite, and ileal expression and plasma levels of satiety hormones such as insulin, ghrelin, cholecystokinin, and glucagon‐like peptide 1 (Chungchunlam, Henare, Ganesh, & Moughan, 2015; McAllan, D Cotter, & M Roche, 2012; Pal, Radavelli‐Bagatini, Hagger, & Ellis, 2014). Additionally, in several studies in which whey protein constituted the main protein source of a high‐fat diet (HFD), this protein ameliorated the impairment of glucose tolerance, and reduced the increase in body weight and adiposity compared to other dietary proteins, such as casein (CAS, a nonwhey milk protein which is the main component of cheese), soy, meat, and fish proteins (Madsen, Myrmel, Fjære, Liaset, & Kristiansen, 2017; McAllan et al., 2013; Pilvi, Korpela, Huttunen, Vapaatalo, & Mervaala, 2007). Whey protein also influences lipid metabolism in adipocytes, including plasma release of triacylglycerol (TAG), and reduce intestinal fat absorption (McAllan et al., 2013; Pal, Ellis, & Dhaliwal, 2010; Stanstrup, Schou, Holmer‐Jensen, Hermansen, & Dragsted, 2014). For these reasons, whey protein has been chosen as a good candidate for dietary interventions aiming to prevent or ameliorate metabolic diseases such as obesity.

Obesity is a complex and multifactorial disease characterized by an excess of adiposity due to an imbalance between energy intake and energy expenditure (De Lorenzo, 2016). According to the World Health Organization, in 2016, 1.9 billion adults worldwide were overweight or obese of which 650 million had obesity. It is well known that metabolic disorders are linked with other metabolic diseases, such as cardiovascular diseases, diabetes, adipose tissue dysfunction, as well as a dysbiosis of the intestinal microbial population (Carding, Verbeke, Vipond, Corfe, & Owen, 2015). The gut microbiota plays an important role in the modulation of the host metabolism and in energy harvesting through fermentation of fibres and proteins from food that were not digested and absorbed within the small intestine. Some studies have shown that low‐fat diet (i.e., LFD) and HFD containing whey protein isolate (WPI) have similar impacts on the gut microbiota, increasing the abundance of the families *Bifidobacteriaceae* and *Lactobacillaceae*, and *Bifidobacterium spp.,* and *Lactobacillus spp*. However, these studies were performed with in vitro gastrointestinal digestion models or 16S rRNA compositional metagenomic approaches and, thus, there are no studies describing in detail which species and which microbial functions are influenced by the presence of WPI in the diet (McAllan et al., 2014; Sánchez‐Moya et al., 2017; Sprong, Schonewille, & van der Meer, 2010).

Other factors that considerably shape the gut microbiota in humans in early stage of life of the individual are modality of delivery and genetics, which influence the microbial colonization in the perinatal and early‐postnatal period. The microbiota evolves and becomes more similar to an adult microbial configuration by the end of 3–5 years of life. From this stage, the gut microbiota becomes relatively stable throughout life, but factors such as diet, antibiotic treatment, lifestyle, and bacterial infections can strongly affect and deviate the gut microbiota stability. In turn, this can have an impact on host metabolism and host physiology (O'Toole & Jeffery, 2015; Rodriguez, 2015). In contrast to the human case, the link between age and microbiota in rodents is still poorly understood. A recent study carried out in rodents, particularly in healthy Sprague Dawley rats, revealed a significant change in the composition of their microbiota from 3 weeks to 2 years of age, with consequences on the microbial fermentative capacity and thus, host metabolism regulation (Flemer et al., 2017). Notably, we have shown that WPI intake (20% energy) starting from early adolescence and continuing for 8‐week period reduces weight gain and adiposity (McAllan et al., 2013), but extending intake into 21 weeks (and hence as mice get older), there is an impairment of the effects of WPI (McAllan et al., 2014). These data suggested a potential interplay between duration of WPI and HFD intake on parameters related to energy balance that appear to be sensitive to the animals age. Untangling the relative importance of each for energy balance regulation is difficult to achieve because duration of the diet intake and age go together, instead one can focus on how different dietary component interplay with age by fixing one dietary component and changing another, and then assess the influence on energy balance. This would provide the basis for development of better interventions for prevention of weight gain and development of obesity in individuals at risk as they grow from young to older age.

**Figure 1:**
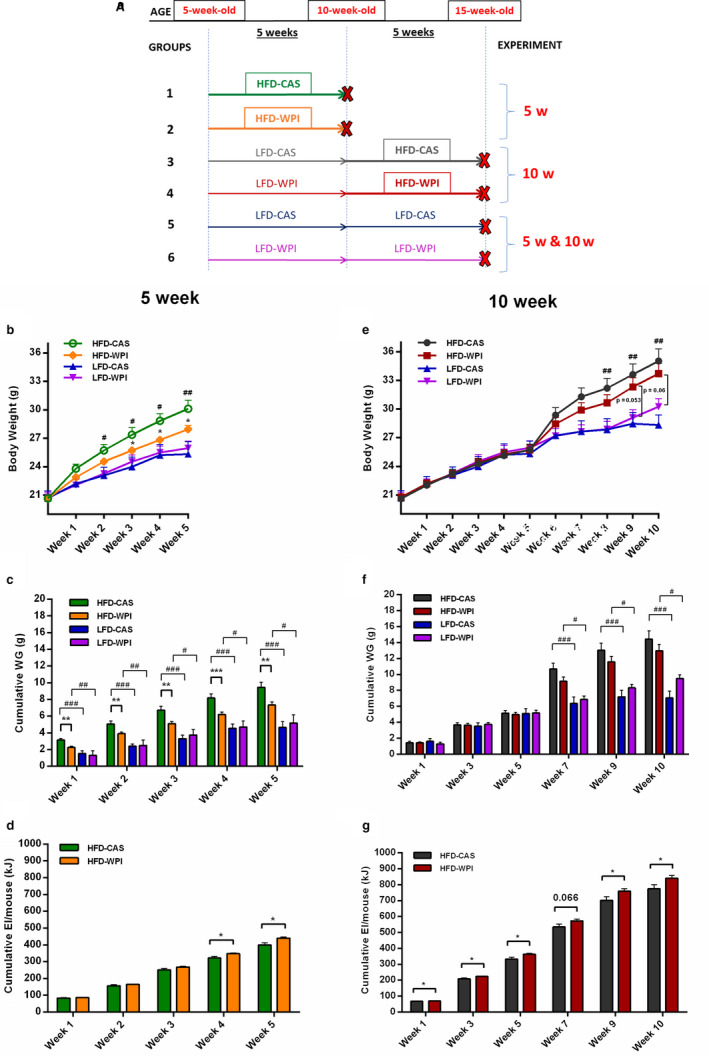
Effect of HFD‐WPI on body weight and energy intake in 5w versus 10w mice. (a) Mice were divided into six groups. 5‐week‐old mice (5 w, groups 1 and 2) were fed a high‐fat diet with control casein (HFD‐CAS; 45% fat and 20% casein) or whey protein isolate (HFD‐WPI; 45% fat and 20% whey protein isolate). As controls for 5w, groups 5 and 6, respectively, were fed a low‐fat diet with casein (LFD‐CAS; 10% fat and 20% casein) or whey protein isolate (LFD‐WPI; 10% fat and 20% whey protein isolate). Groups 3 and 4 (10 w) were initially fed with LFD‐CAS and LFD‐WPI respectively. After 5 weeks, when they were 10‐week‐old, the diet of these groups was switched from LF to HF, which lasted for other 5 weeks. The groups 5 and 6 (i.e., LFD‐CAS and LFD‐WPI) represented the control also for 10w. The present data show (b) body weight and (c) cumulative weight gain of 5w mice fed with HFD‐CAS, HFD‐WPI, LFD‐CAS, and LFD‐WPI. In panel (d) is indicated cumulative energy intake of 5w mice fed with HFD‐CAS and HFD‐WPI. Data also show (e) body weight and (f) cumulative weight gain intake of 10w mice fed with HFD‐CAS, HFD‐WPI, LFD‐CAS, and LFD‐WPI. In panel (g) is indicated cumulative energy of 10w mice fed with HFD‐CAS and HFD‐WPI. *Statistical analysis*: in figure (b) and (e) groups showing * (for HFD‐CAS vs. HFD‐WPI) and # (HFD‐CAS vs. LFD‐CAS) are significant (*/#*p* < .05 or **/##*p* < .01 or ***/###*p* < .001). In figure (e), the trends refer to HFD‐WPI vs. LFD‐WPI. In Figure 1 (c), (d), (f) and (g) groups showing * (for HFD‐CAS vs. HFD‐WPI and LFD‐CAS vs. LFD‐WPI) and # (HFD‐CAS vs. LFD‐CAS and HFD‐WPI vs. LFD‐WPI) are significant (*/#*p* < .05 or **/##*p* < .01 or ***/###*p* < .001). A complete statistical description is detailed in Methods and Materials and “Supplementary Statistics”

In the present study, we aimed to investigate the relationship between WPI‐supplemented diet duration, age, microbiome and host energy balance, using 5‐week and 10‐week‐old C57BL/6J mice. To assess these interactions, we fixed HFD to a limited period (5 weeks) in young and older mice. We have previously shown that feeding high (40% by energy) WPI as part of a 45% energy HFD to adolescent mice was shown to reduce weight gain, with animals attaining a lean phenotype but where the microbial composition was distinct to LFD fed mice (McAllan et al., 2014). This led to our first hypothesis that WPI intake as part of LFD during adolescence would bring about the microbial and endocrine changes necessary to impede weight gain resulting from a subsequent switch to a HFD in older mice but while continuing to ingest WPI. Because microbial changes in the gut occurs early life, and it was not clear at what stage in adolescence the change in microbial composition occurs in relation to WPI intake, we also assessed the impact of WPI as part of a HFD much earlier on in adolescence (younger mice) without a pre‐intervention with LFD containing WPI. To this end, we focus mainly on the effects of a HFD containing a standard amount of WPI (20% energy) on body weight, energy intake, plasma hormones, metabolites and genes encoding nutrient transporters, lipid metabolism enzymes, and hormones in the ileum and in the adipose tissue in mice at two different stages of life (i.e., adolescence, from week 4 to week 8–9 of life, and early adulthood, from week 9 to 17–18 of life) (Brust, Schindler, & Lewejohann, 2015). In addition, using a high‐throughput shotgun metagenomic approach, we performed a gut microbiota analysis on faecal samples. Unlike the 16S rRNA gene sequencing approach, shotgun metagenomics allows a more detailed view of the microbial composition and genes and metabolic pathways related to the microbial community (Quince, 2017). Specifically, we performed a first experiment with 5‐week diet duration (i.e., 5w) in which 5‐week‐old mice were fed a HFD, containing WPI (HFD‐WPI) or the control CAS (HFD‐CAS) as the protein source. In parallel, we performed a second experiment with 10 week‐diet duration (i.e., 10w), in which 5‐week‐old mice were fed LFD‐CAS or LFD‐WPI for 5 weeks and, when they were 10‐weeks old, their diet was switched to HFD‐CAS or HFD‐WPI for a further 5 weeks. The control groups were maintained on LFD‐CAS or WPI for the entirety of the experiment (Figure 1a). The above experimental design allowed us to fix the HFD feeding duration to 5 weeks in both experiments in younger and older mice, and focus on the potential interplay of the effects of WPI duration and age (shorter duration in younger mice in experiment 1 versus longer duration as mice age in experiment 2).

We predicted that: (a) giving WPI in LFD during a pre‐HFD period (i.e., second experiment 10w) would prevent/ameliorate the metabolic syndrome outcomes that would occur after switching to HFD and extending the WPI supplementation; (b) energy balance, adipose tissue metabolism, gut microbiota, and nutrient absorption would exhibit differences due to the dietary intervention carried out at two different stages of life (i.e., both experiments, 5w and 10w).

## 2 METHODS AND MATERIALS

### 2.1 Experimental strategy

The *in vivo* experiments were approved by the University College Cork Animal Experimentation Ethics Committee (2015/007), were licenced under the European Directive 2010/63/EU and comply the ARRIVE guidelines. Eighty C57BL/6J 3‐week‐old male mice were purchased commercially (Harlan; UK) and were housed 4 per cage on a 12 hr light/dark cycle. The mice had ad libitum access to food and water throughout the study unless otherwise stated. During the initial 2 weeks of acclimatisation period, mice were provided with a diet containing 10% low fat diet and 20% casein (LFD‐CAS; #D12450B; Research Diets, USA; % by values of energy). Subsequently, weight matched mice received different diets varying only in protein quality or fat content (Research Diets, USA): (1) LFD‐CAS, (2) 10% fat and 20% whey protein isolate (LFD‐WPI; #D1208/601), (3) 45% fat and 20% casein (HFD‐CAS; #D12451) and (4) 45% fat and 20% whey protein isolate (HFD‐WPI; #D11040501). The composition of each diet is detailed in the Table T1.

**Table T1:** XXXX

From Research Diets, USA	LFD‐CAS	LFD‐WPI	HFD‐CAS	HFD‐WPI
	#D12450B	#D1208/601	#D12451	#D11040501
fat % (by values of energy)	10	10	45	45
CAS or WPI % (by values of energy)	20	20	20	20

Ingredients	gm	Kcal	gm	Kcal	gm	Kcal	gm	Kcal
Casein	200	800	0	0	200	800	0	0
Whey Protein	0	0	200	800	0	0	200	800
L‐Cysteine	3	12	3	12	3	12	3	12
Corn Starch	315	1,260	315	1,260	72.8	291	72.8	291
Maltodextrin 10	35	140	35	140	100	400	100	400
Sucrose	350	1,400	350	1,400	172.8	691	172.8	691
Cellulose, BW200	50	0	50	0	50	0	50	0
Soybean Oil	25	225	25	225	25	225	25	225
Lard*	20	180	20	180	177.5	1598	177.5	1598
Mineral Mix S10026	10	0	10	0	10	0	10	0
DiCalcium Phosphate	13	0	13	0	13	0	13	0
Calcium Carbonate	5.5	0	5.5	0	5.5	0	5.5	0
Potassium Citrate, 1 H2O	16.5	0	16.5	0	16.5	0	16.5	0
Vitamin Mix V10001	10	40	10	40	10	40	10	40
Choline Bitartrate	2	0	2	0	2	0	2	0
Total	1,055	4,057	1,055	4,057	858.1	4,057	858.1	4,057

Two experiments were done:

#### 2.1.1 Experiment in which mice started the HFD dietary intervention at 5‐week‐old (5w)

5‐week‐old mice were provided HFD‐CAS or HFD‐WPI (4 cages per group, each with 4 animals) and LFD‐CAS or LFD WPI (controls; 2 cages per group, each with 4 animals) for 5 weeks. After 5 weeks of dietary intervention, only the 10‐week‐old animals fed with HFD (groups 1 and 2) were euthanized (Figure 1a).

#### 2.1.2 Experiment in which mice started the HFD dietary intervention at 10‐week‐old (10w)

5‐week‐old mice were provided LFD‐CAS or LFD‐WPI for 5 weeks and switched to HFD‐CAS or HFD‐WPI for further 5 weeks (four cages per group and four animals per cage). The controls were the same animals as the experiment 5w, that is, mice fed with LFD‐CAS or LFD‐WPI (two cages per group, each with four animals) for 10 weeks. At the end of the dietary intervention, all the 15‐week‐old animals (groups 3, 4, 5, and 6) from the four groups (i.e., HFD‐CAS, HFD‐WPI, LFD‐CAS, and LFD‐WPI) were euthanized (Figure 1a).

In both experiments body weight was measured weekly, as well as the food intake per cage and was converted in energy (using the energy density HFD = 4.73 kcal/g and LFD = 3.85 kcal/g of the rodent diets; Research Diets, USA). Notably, in the results are reported energy intake data for HFD‐fed mice and not for LFD‐fed mice because the latter groups had a sample size of 2, thus too small to carry out appropriate statistical analysis. For this reason, we omitted energy intake data for LFD‐fed mice.

The faecal samples were collected from individual mice the day before euthanasia and these were stored at −80°C for subsequent analysis. In both experiments, mice were fasted for 8h commencing at 22.00 in the dark phase, then anaesthetized (100 mg/kg Ketamine and 10 mg/kg Xylazine). Mice were euthanized by cervical dislocation and blood samples and tissues were collected. Tissues weight and/or length were recorded, and the samples were stored at −80°C for subsequent analysis.

### 2.2 RNA extraction and gene expression analysis

Total RNA was extracted from the ileum and liver (approximately 30 mg of tissue per each sample) using RNeasy Minikit and QIAshredder columns (Qiagen), and from epididymal white adipose tissue (eWAT) and subcutaneous white adipose tissue (sWAT) using QIAzol Lysis Reagent (Qiagen). The extracted RNAs were treated with DNase (Qiagen, Ireland). Complementary DNA was synthetized from 600ng total RNA using SuperscriptTM II Reverse Transcriptase kit (Life Technologies, Ireland), and subjected to Real‐Time PCR (Roche, Ireland) using SYBR Green Select Master Mix (Roche, UK) as detailed before (McAllan, Speakman, Cryan, & Nilaweera, 2015). The gene expression was calculated using 2‐ΔΔCp and normalised against the reference genes *β‐actin* (intestine and adipose tissue) and *Gapdh* (liver), and presented as a ratio versus average of HFD‐CAS 5w. The sequence of the primers is detailed in Table ST1. RNA extraction and gene expression analysis were carried out in HFD‐CAS and HFD‐WPI‐fed mice in both experiments (i.e., 5w and 10w), but not in the LFD control groups.

**Table ST1:** XXX

Genes	Forward primer (5′‐3′)	Reverse primer (5′‐3′)
Acetyl‐CoA carboxylase *(Acc1)*	5′‐cagtgctatgctgagattgagg‐3′	5′‐acacagccagggtcaagtg‐3′
Fatty acid transporter 1 (*Fatp1)*	5′‐ccggtgtggtggctgctcttctc‐3′	5′‐gctgccatctccccgccataaatg‐3′
Fatty acid synthase (*Fasn*)	5′‐gctgctgttggaagtcagc‐3′	5′‐agtgttcgttcctcggagtg‐3′
Lipoprotein lipase (*Lpl*)	5′‐tgtctaactgccacttcaaccac‐3′	5′‐gggcacccaactctcatacattc‐3′
Cluster of differentiation 36 (*CD36*)	5′‐ttgaaaagtctcggacattgag‐3′	5′‐tcagatccgaacacagcgta‐3′
Leptin (*Ob*)	5′‐ccccgcaccgctggaagtac‐3′	5′‐atgtgccctgaaatgcggtatg‐3′
Cluster of differentiation 68 *(CD68)*	5′‐cacttcgggccatgtttctcttg‐3′	5′‐aggggctggtaggttgattgtcgtc‐3′
Carnitine palmitoyltransferase 1a (*Cpt1a*)	5′‐gactccgctcgctcattc‐3′	5′‐tctgccatcttgagtggtga‐3′
Carnitine palmitoyltransferase 1b (*Cpt1b*)	5′‐gagtgactggtgggaagaatatg‐3′	5′‐gctgcttgcacatttgtgtt‐3′
Hormone‐sensitive lipase (*Hsl*)	5′‐ctattcagggacagaggcag‐3′	5′‐cgatgtggtcttttggggc‐3′
Uncoupling protein 2 (*Ucp2*)	5′‐ccatttcctgcaccccgatttacttcc‐3′	5′‐gctgggctggggatgaagatgaag‐3′
Uncoupling protein 3 (*Ucp3*)	5′‐acaggcccacacggtccagaacc‐3′	5′‐cccatcaggtcagtgcaaaacagagg‐3′
Angioprotein‐like 4 (*Fiaf*)	5′‐gctcattggcttgactcccaac‐3′	5′‐aaaagtccactgtgccgctc‐3′
Glucose transporter 2 (*Glut2*)	5′‐tcctacttggcctatctgctgtgc‐3′	5′‐tgccctgacttcctcttccaac‐3′
Fatty acid transporter 4 (*Fatp4*)	5′‐tggcgtttcatccgggtcttcatc‐3′	5′‐gcaaacagcaggggcaccgtcttc‐3′
L‐type amino acid transporter 4 (*Lat4*)	5′cccgcttcctgttgttggtgctaac3′	5′ggggcttcttctcaggctttcaag 3′
Sodium‐glucose co‐transporter 1 (*Sglt1*)	5′‐gagccccgcggttactgc‐3′	5′‐cctgcggctgctcctgtg‐3′
System B(0) neutral amino acid transporter 1 (*Slc6a19*)	5′gtgtggcgcttcccctacctatg‐3′	5′ cctctgaccgatggcaaactcc‐3′
Peptide YY (*Pyy*)	5′‐ggacgcctaccctgccaaacca‐3′	5′‐agtgccctcttcttaaaccaaaca‐3′
Proglucagon (*Cgc*)	5′‐agggacctttaccagtgatgtga‐3′	5′‐acgagatgttgtgaagatggttgt‐3′
Malonyl CoA‐acyl carrier protein transacylase (*Mcat*)	5′‐cagtgtgggagagtttgctg‐3′	5′‐ccttcaccgcatacagacc‐3′
Stearoyl‐CoA desaturase *(Scd1)*	5′‐ttccctcctgcaagctctac‐3′	5′‐cagagcgctggtcatgtagt‐3′
Glyceraldehyde 3‐phosphate dehydrogenase (*Gapdh*)	5′‐aagagggatgctgcccttac‐3′	5′‐ccattttgtctacgggacga‐3′
β‐actin (*ActB*)	5′‐agagggaaatcgtgcgtgac‐3′	5′‐caatagtgatgacctggccgt‐3′

### 2.3 Leptin, glucose, insulin, and triacylglycerol levels

Plasma leptin and glucose levels were determined using Mouse Leptin ELISA kit (Crystal Chem, USA) and Mouse Glucose Assay (Crystal Chem, USA), respectively. Mouse Insulin ELISA Kits, Ultra‐Sensitive Assay (Crystal Chem, USA) was used to determine the plasma insulin level.

The weights of liver and previously dried caecum content samples were recorded, and the samples were homogenised in 1,5 ml of %NP40/ddH2O solution. After two repeated steps of heating (80°C–100°C for 3–5 min) and cool down (15 min at RT), all the samples were centrifuged at maximum speed. The supernatant, containing the lipids, was collected and diluted 1:10. Triacylglycerol (TAG) level in the plasma, in the liver extract and in the caecum content extract it was measured using Triglyceride Quantification Assay Kit (abcam, UK).

Leptin, glucose, insulin, and TAG levels detection were carried out in all the groups, in both experiments.

### 2.4 DNA extraction and library preparation

Genomic DNA from faecal samples collected from HFD groups at the end of experiment 5w and 10w was purified according to the protocol for the standard Qiagen QiaAMP DNA Fast Stool Kit (Qiagen, Germany). Total DNA was accurately quantified with Qubit dsDNA HS Assay kit (Bio‐Sciences, Dublin, Ireland).

After DNA extraction, samples were processed for shotgun metagenomic sequencing using the Nextera XT DNA Library Prep Kit (Illumina). After passing quality checks, libraries were prepared with Nextera XT DNA library prep kits (Illumina), the samples were sequenced on the Illumina MiSeq platform (2 × 250 bp paired‐end reads) in the Moorepark Teagasc sequencing facility, following standard Illumina sequencing protocols (Illumina 2013). DNA extraction and library preparation were carried out in HFD‐CAS and HFD‐WPI‐fed mice in both experiments (i.e., 5w and 10w), but not in the LFD control groups.

### 2.5 Bioinformatic analysis (shotgun metagenomic sequencing data)

Metagenomic raw sequence reads were filtered on the basis of quality (removal all bases at the 3’ end with an average Phred score <25 over a sliding window of 10 bp) and length (only reads with a minimum length of 180 bases were considered for further analysis) with PRINSEQ‐lite 0.20.4 (Schmieder & Edwards, 2011). After filtering, the sequences from the host, in our case the mouse, were eliminated. The mouse genome (Mus musculus (Mouse) GRCm38) downloaded from llumina iGenomes (https://support.illumina.com/sequencing/sequencing_software/igenome.html) was use as reference to remove host reads using BMTagger (Version 3.101) (Rotmistrovsky & Agarwala, 2011). Paired‐end reads were joined and filtered for duplicates using Picard tools 2.7.1, specifically FastqToSam and MarkDuplicates tools respectively (“Picard Toolkit.” 2019. Broad Institute, GitHub Repository. http://broadinstitute.github.io/picard/). Subsequently, the reads underwent another stage of quality filtering, sub‐quality, using a modified version of the script trimBWAstyle.pl that works directly from BAM files (TrimBWAstyle.usingBam.pl.2010;https://github.com/genome/genome/blob/master/lib/perl/Genome/Site/TGI/Hmp/HmpSraProcess/trimBWAstyle.usingBam.pl). This script was used to delete reads with a Quality threshold value less than Q30. In addition, the reads shorter than 200 bp were also eliminated.

The analysis of the microbial composition was carried out using the MetaPhlAn2 species classifier (Segata et al., 2012). Taxa with a relative abundance of <0.01% were categorized as "Other" for each classifier. Subsequently, the functions were assigned with HUMAnN2 tool (Franzosa et al., 2018), based on ChocoPhlan and UniRef databases (Suzek, Huang, McGarvey, Mazumder, & Wu, 2007). The HUMAnN2 gene abundance table was regrouped by a mapping of GO terms for all categories of bacterial metabolism and dividing the functional table into two files (one stratified and one nonstratified). Metagenome assembly was performed using IDBA‐UD (Peng, Leung, Yiu, & Chin, 2012).

### 2.6 Statistical analysis

Power and coefficient of variation were calculated using data (body weight gain) from a previous study (McAllan et al., 2015) and determined to be 8.8%. For a power of 80% and a significance level of 5%, this allows detection of a difference of 14% with a sample size of at least 8 mice per treatment. Body weight, cumulative weight gain, and cumulative energy intake differences over both experiments 1 and 2 were analyzed by a two‐way repeated‐measures ANOVA with Bonferroni's *post hoc* pairwise comparisons. Statistical analysis of tissue weight, intestine weight/intestine length, gene expression, plasma leptin, insulin, glucose and TAG, and caecal and hepatic TAG data were performed using two‐way ANOVA followed by pairwise comparison using Bonferroni's *post hoc* test. Nonparametric data were compared by Kruskal–Wallis ANOVA followed by Mann‐Whitney *U* test. Tissues weight was also evaluated using ANCOVA. Data were expressed as mean ± *SEM* and significance was set at *p* < .05 using SPSS software version 24 (IBM Corp). A complete description is detailed in “Supplementary Statistic” and Figures S4 and S5.

Bacterial and archaeal reads were retrieved from the output of MetaPhlAn2 for taxonomic profiling. Functional assignment was performed at the GO gene level. Statistical analyses were carried out with R (R version 3.4). Different R’s packages have been used (R Development Core Team 2016). To perform alpha diversity analyses (Richness, Shannon Index and Simpson Index), as well as the multidimensional scaling analysis (MDS) based on Bray‐Curtis, the statistical package “vegan” version 2.3.0 (Oksanen et al.,) was used. The Adonis function in “vegan” was used for PERMANOVA (permutational analysis of variance) analysis, and betadisper function for MDS figures. To compare alpha diversity metrics among groups, ANOVA (aov) test was performed and it was adjusted with the Benjamini–Hochberg method. Statistical differences between multiple samples in taxonomic and functional analyses were estimated by Kruskal–Wallis and False discovery rate (FDR). The Benjamini–Hochberg procedure was used to correct for multiple testing, and differences between two samples were estimated by Mann–Whitney test analysis and corrected with Family‐wise error rate. The ggplot2 package (version 3.2.0) was used for data visualization (2016).

Statistical significance was established at *p* < .05 (*), with other ranges, that is, *p* < .01 (**) and *p* < .001 (***), *p* < .0001 (****), based on the corrected p‐values, also being noted.

## 3 RESULTS

### 3.1 HFD containing WPI increased energy intake but decreased body weight gain in younger mice

During the first 5 weeks (5w, Figure 1a), 5‐week‐old mice fed HFD‐CAS significantly increased body weight starting from week 2 compared to the LFD‐CAS (Figure 1b). On the contrary, mice fed with HFD‐WPI did not differ from their control group (i.e., LFD‐WPI) for the duration of the experiment. HFD‐WPI‐fed mice exhibited significantly lower body weight, from week 3, and cumulative weight gain, from week 1, compared to HFD‐CAS‐fed mice (Figure 1b,c). During the experiment, not just HFD‐CAS‐fed mice, but also HFD‐WPI‐fed mice, had significantly higher cumulative body weight gain compared to LFD groups at every time point (Figure 1c). The energy intake did not match the body weight and cumulative weight gain data: the HFD‐WPI‐fed mice respectively showed an increase in the cumulative energy intake compared to CAS counterpart (week 5 time point, HFD‐CAS vs. HFD‐WPI: 400.70 ± 12.27 kJ vs. 440.07 ± 6.90 kJ; Figure 1d).

In contrast, HFD containing WPI given to 10‐week‐old mice (10w, Figure 1a) produced different outcomes with respect to energy balance compared to HFD‐WPI feeding in younger individuals. LFD‐fed mice supplemented with WPI or CAS had similar body weight trajectories until week 5, similar to our previous study (Figure 1b) (Nilaweera et al., 2017). When all the mice were 10‐week‐old, switching the diet to HF significantly increased body weight and cumulative weight gain of HFD‐CAS group from week 7 compared to LFD‐CAS control group (Figure 1e,f). In this instance, the body weight trajectory of HFD‐WPI‐fed mice was similar to the LFD‐WPI‐fed mice until week 9 (Figure 1e), but their cumulative weight gain was higher starting from week 7 compared to LFD counterparts (Figure 1f). Unlike 5w mice, in 10w mice no difference between HFD‐CAS and HFD‐WPI body weight trajectories and cumulative weight gain were observed (*p* > .05) (Figure 1e).

During 10w, in accordance 5w, the WPI groups fed with HFD showed a significant increase in the cumulative energy intake relative to CAS group (week 10 time point, HFD‐CAS vs. HFD‐WPI: 774.85 ± 26.10 kJ vs. 841.50 ± 16.68 kJ) (Figure 1g). Clearly, this indicates that a loss of energy occurs during WPI supplementation.

To investigate the aforementioned loss of energy, we quantified the level of lipids, more specifically triacyclglycerols, in the caecum, but did not observe any differences between CAS and WPI groups except in the 10w HFD‐WPI group, which showed a trend toward a decrease compared to 10w HFD‐CAS (Figure S1a and S1b). In addition, 10w HFD groups showed a significantly higher amount of lipids in the caecum compared to 5w HFD groups (Figure S1a). This analysis did not yield an explanation for the discrepancy of energy between WPI and CAS groups, and the underlying cause remains unclear.

### 3.2 Effect of HFD‐WPI on organs and tissues at different ages

**Figure 2:**
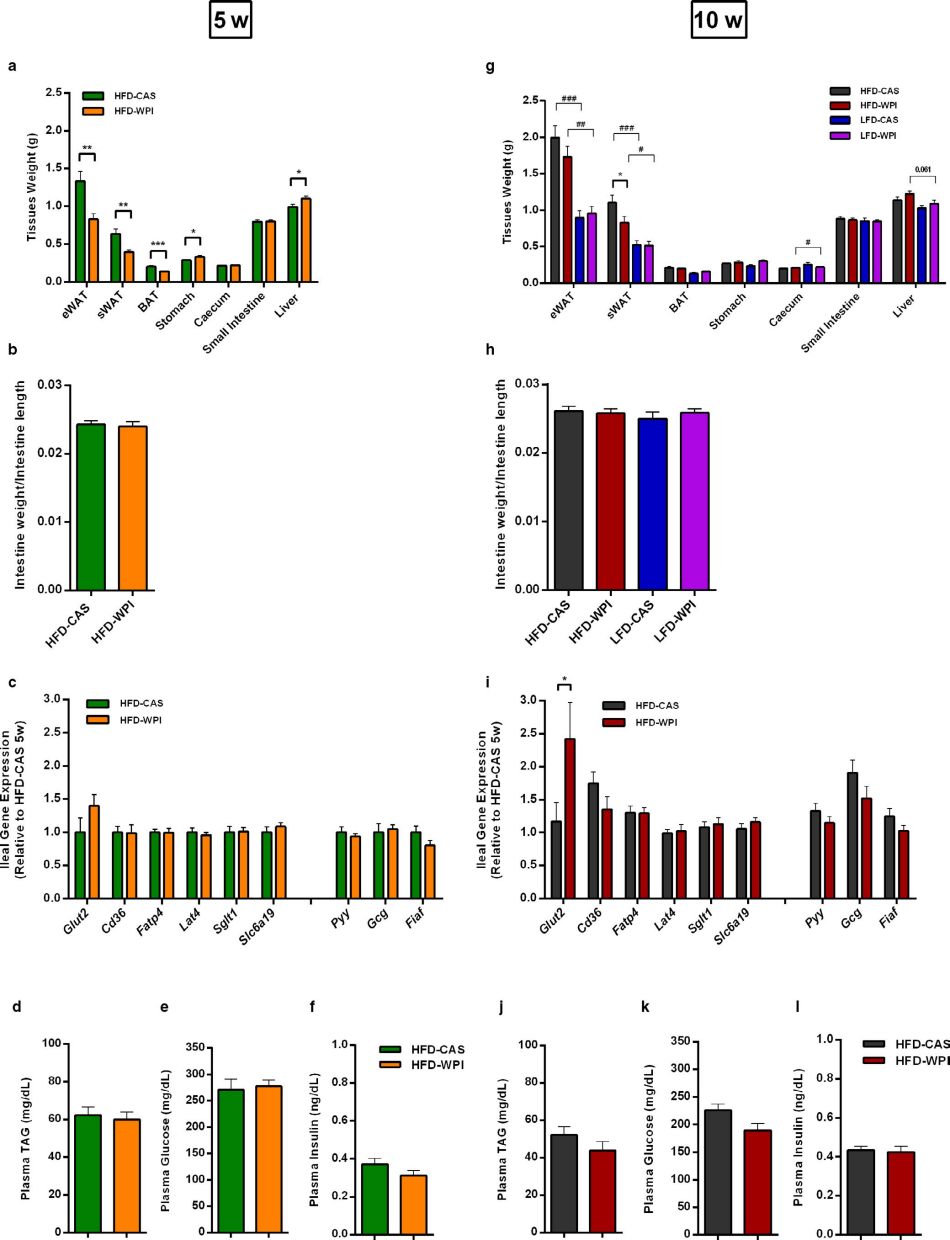
HFD‐WPI effect on tissues and organs weight, ileal gene expression and plasma metabolites and insulin in 5w vs. 10w mice. Data show (a) the tissues and organs absolute weight, (b) intestine weight normalized by intestine length, (c) ileal gene expression of hormones and nutrient transporters, plasma levels of (d) triacylglycerol (TAG), (e) glucose and (f) insulin of 5w mice fed with HFD‐CAS and HFD‐WPI. Also shown are (g) the tissues and organs absolute weight, (h) intestine weight normalized by intestine length, (i) ileal gene expression of hormones and nutrient transporters, plasma levels of (j) triacylglycerol (TAG), (k) glucose and (l) insulin of 10w mice fed with HFD‐CAS and HFD‐WPI. Abbreviation; eWAT; epididymal white adipose tissue, sWAT; subcutaneous white adipose tissue, BAT; brown adipose tissue, GLUT2; glucose transporter 2, CD36; cluster of differentiation 36, FATP4; fatty acid transporter 4, LAT4; L type amino acid transporter 4, SGLT1; sodium‐glucose transporter 1, SLCa19; methionine transporter, PYY; peptide YY, GCG; proglucagon, FIAF; fasting‐inducing adipose factor. *Statistical analysis*: groups showing * (for HFD‐CAS vs. HFD‐WPI) and # (HFD‐CAS vs. LFD‐CAS and HFD‐WPI and LFD‐WPI) are significant (*/#*p* < .05 or **/##*p* < .01 or ***/###*p* < .001). A complete statistical description is detailed in Methods and Materials and in figures S4 and S5

Mice that received HFD‐WPI at 5‐week‐old decreased the weight of adipose tissues (i.e. sWAT, eWAT and BAT) and increased liver and stomach weights, compared to the HFD‐CAS group (Figure 2a). No differences in caecum and small intestine weights were observed between the two groups (Figure 2a). In addition, an organ/tissues weights evaluation using ANCOVA (body weight as covariant) was also performed, which also yielded similar results (see supplementary statistics Figure S5b and S5c). Details of tissue weight at this time point in the LFD‐CAS and LFD‐WPI have previously been detailed elsewhere (Nilaweera et al., 2017).

Prolonged WPI intake as part of LFD (10w mice) had no effect on organs and tissues weights (Figure 2g). However, when WPI‐fed mice and their controls were exposed to HFD at 10‐week‐old, they had increased eWAT and sWAT weights compared to LFD‐fed mice (Figure 2g). While there were no differences observed in eWAT and BAT weight between HFD‐CAS and HFD‐WPI‐fed mice, sWAT and was significantly lower for 10w mice fed with HFD‐WPI than those fed with HFD‐CAS (Figure 2g). No major changes were observed in stomach, caecum, small intestine, and liver across the groups (Figure 2g).

The small intestine length (data not shown) and intestinal weight normalized to the length were similar across both 5w and 10w mice (Figure 2b,h).

From the organs weight data, we noticed that the weight of the liver was higher in mice fed a WPI (even if in 10w mice this difference did not achieve the significance). To investigate the possible reason behind this finding, we measured the level of gene expression of selected enzymes involved in hepatic fatty acids biosynthesis, namely acetyl‐CoA carboxylase (*Acc1*), fatty acids synthase (*Fasn*), malonyl CoA‐acyl carrier protein transacylase (*Mcat*), and acyl‐CoA desaturase 1 (*Scd1*). To carry out this analysis, we solely focused on HFD‐fed mice, both 5w and 10w. While no changes between HFD‐CAS and HFD‐WPI (both 5w and 10w) were observed in the gene expression of *Acc1*, *Fasn,* and *Mcat*, the expression level of the gene *Scd1* was lower in both HFD‐WPI 5w and 10 weeks compared to the CAS counterparts (Figure S2a). SCD1 takes part in TAG biosynthesis and animals with a deficiency in SCD1 have a lower rate of TAG biosynthesis (Flowers & Ntambi, 2008). According to these results, we found that 5w mice showed a lower level of hepatic TAG in the presence of WPI. However, no differences in liver TAG were detected between 10w mice (Figure S1c). This last result disagrees with *Scd1* gene expression data. In addition, hepatic TAG of both 10w mice were similar to HFD‐WPI 5w mice and lower than HFD‐CAS 5w mice (Figure S1c). No statistical difference in hepatic TAG has been observed between LFD‐CAS and LFD‐WPI fed mice (Figure S1d).

In summary, the reason why the liver is heavier during WPI supplementation is not because of a hepatic TAG accumulation. Thus, the underlying cause remains to be elucidated.

### 3.3 HFD‐WPI does not influence ileal gene expression, glucose, and insulin plasma levels

Considering the differences observed in the body/organs weight and energy intake data across the different groups, we investigated further by measuring the ileal expression level of genes involved in the transport of nutrients and genes encoding for proteins involved in energy balance metabolism. We focused most of our analysis specifically on mice that received HFD‐CAS and HFD‐WPI at both ages (i.e., Figure 1a groups 1, 2, 3 and 4), as differences in mice fed with LFD‐CAS or WPI have been previously investigated (McAllan et al., 2013, 2014, 2015).

WPI intake during 5w had no significant impact on the ileal expression of nutrient transporters and satiety hormones in HFD mice (Figure 2c,i). In contrast, adults fed a HFD‐WPI increased glucose transporter 2 (*Glut2*) expression compared to HFD‐CAS‐fed mice (Figure 2i). Furthermore, fatty acids transporter (*Cd36*) expression increased in the 10w HFD‐CAS group compared to the 5w HFD‐CAS group, without showing any differences between the WPI groups, and the gene encoding fatty acids transporter *Fatp4* was significantly more expressed in both 10w groups, compared to the 5w groups (Figure S2b). No differences were detected in the amino acid transporters (i.e., *Lat4* and *Slc6a19*) and the sodium‐glucose transporter *Sglt1* gene expression across all groups (Figures 2c,i and S2b). In addition, we observed an increase in the expression of anorexic hormone Peptide Tyrosine Tyrosine (*Pyy*) in 10w HFD‐CAS‐fed mice relative to 5w HFD‐CAS‐fed mice, and an increase in *Gcg* (i.e., a gene that codes for the precursor of the satiety hormone GLP‐1 in the intestinal L cells of the ileum) in both 10w HFD groups, compared to 5w HFD groups. The expression of the Fasting‐Induced Adipose Factor (*Fiaf*) remained unchanged (Figure S2b).

Although the expression of fatty acids transporters seems to be higher in older mice, no differences were observed in TAG levels in the plasma between 10w HFD‐CAS and 5w HFD‐CAS‐fed mice and between LFD fed mice (Figures 2d,j, S1e and S1f). On the contrary, TAG levels in the plasma were higher in the 5w HFD‐WPI group relative to the 10w counterpart (Figure S1e).

In addition, no changes were detected between CAS and WPI groups at both ages in glucose and insulin plasma level (Figure 2e,f,k,l). However, 10w HFD‐WPI‐fed mice showed a significant decrease in plasma glucose and an increase in plasma insulin relative to 5w HFD‐WPI‐fed mice. The plasma glucose levels were also lower in 10w HFD‐CAS‐fed mice compared to the 5w counterparts, but insulin remained unchanged between these two groups (Figure S1i and S1j). According to our previous study (McAllan et al., 2015), the two LFD groups had a similar plasma level of insulin (Figure S1j); however, in discordance with the study just mentioned, LFD‐WPI‐fed mice showed lower plasma glucose levels compared to LFD‐CAS‐fed mice (Figure S1h).

These results suggest that the stage of life at which the mice start HFD‐feeding has an influence on glucose and insulin levels and on the expression of genes involved in fatty acids transporters and genes coding for satiety hormones located in the distal part of the small intestine. In this context, the dietary protein typology did not particularly affect our measurements.

### 3.4 HFD‐WPI influences leptin and fat catabolism‐related genes in younger mice

**Figure 3:**
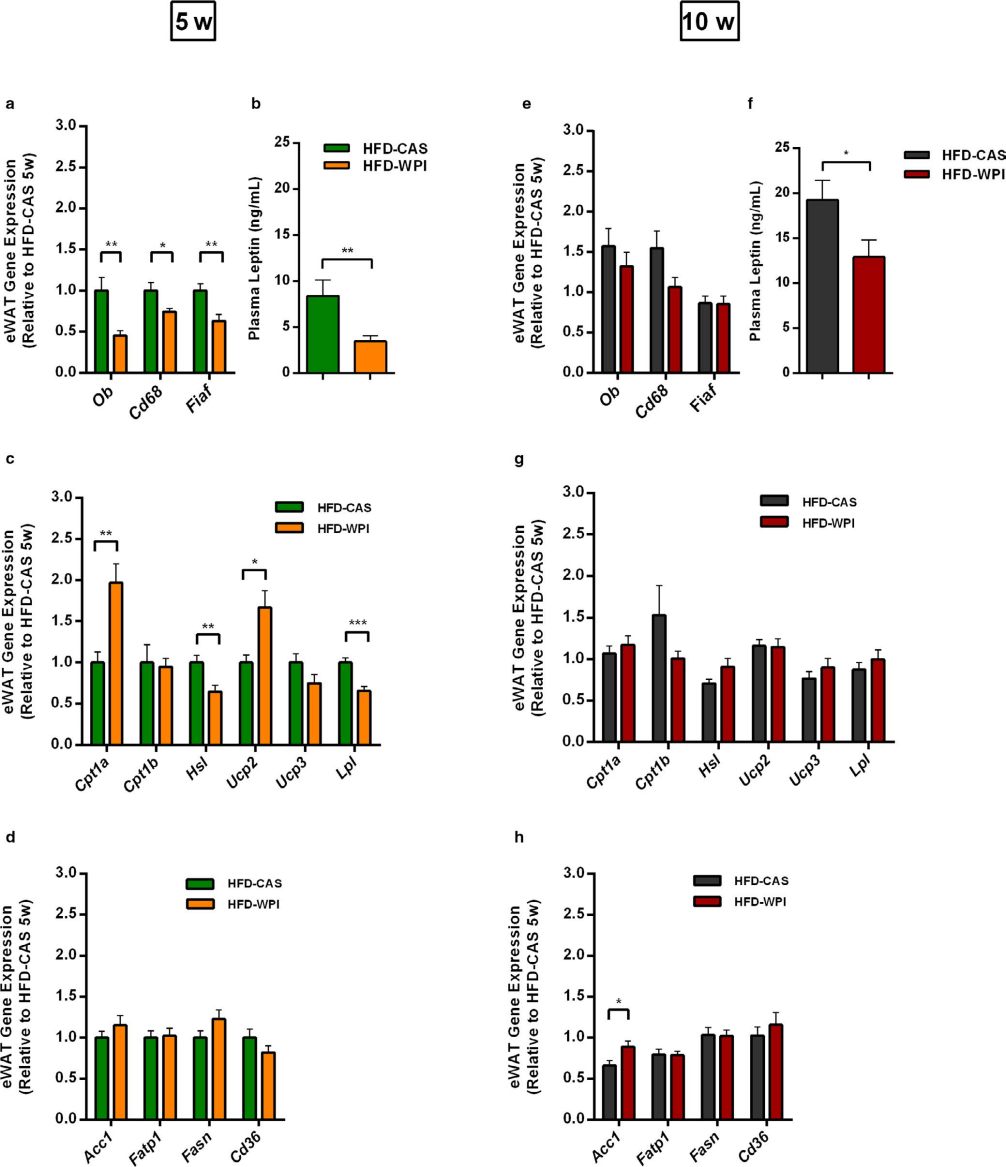
Differential effect of HFD‐WPI on eWAT gene expression and plasma leptin in 5w compared to 10w mice. Data show (a) expression of genes encoding for leptin (*Ob*), inflammation marker CD68 and the FIAF in the epididymal white adipose tissue (eWAT) and (b) plasma levels of leptin of 5w mice fed with HFD‐CAS and HFD‐WPI. Expression of genes encoding for (c) catabolic and (d) anabolic enzymes in the eWAT of 5w mice fed with HFD‐CAS and HFD‐WPI were also investigated. Data also show (e) expression of genes encoding for leptin (*Ob*), inflammation marker CD68 and the FIAF in the eWAT, (f) plasma levels of leptin and expression of genes encoding for (g) catabolic and (h) anabolic enzymes in the eWAT of 10‐week mice fed with HFD‐CAS and HFD‐WPI. Abbreviation; OB; leptin, CD68; cluster of differentiation 68, CPT1a and b; carnitine palmitoyltransferase I, HSL; hormone‐sensitive lipase, UCP2 and 3; uncoupling protein, LPL; lipoprotein lipase, ACC1; acetyl‐CoA carboxylase 1, FATP1; fatty acid transporter 1, FASN; fatty acid synthase, CD36; cluster of differentiation 36. *Statistical analysis*: groups showing * (for HFD‐CAS vs. HFD‐WPI) are significant (*p < .05, **p < .01, ***p < .001). A complete statistical description is detailed in Methods and Materials and Figure S4

A gene expression analysis was also carried out in the eWAT. Interestingly, we observed that *Ob*, *Cd68* (i.e., genes that encode for leptin and for a protein expressed in the monocytes, respectively) and *Fiaf* expressions were significantly lower in the 5w HFD‐WPI‐fed mice relative to the HFD‐CAS control, but this difference was no longer observed between the two 10w groups (Figures 3a,e and S2c). These findings were in accordance with body weight and cumulative weight gain data. Notably, the levels of plasma leptin data matched the *Ob* gene expression levels between the 5w groups (Figure 3b). In older mice, unlike the *Ob* gene expression data, the WPI groups showed a lower level of leptin compared to CAS groups, although this difference was less pronounced compared to the difference between HFD‐CAS and HFD‐WPI in younger mice (Figure 3f). It is also important to highlight that, both *Ob* gene expression and plasma leptin levels were significantly higher in mice that began HFD‐feeding at 10‐week‐old compared to mice that started the HFD intervention at 5‐week‐old (Figures S2c and S1k). No differences in plasma leptin were detected between the LFD groups (Figure S1l).

Analysis of the expression of genes involved in the catabolism within adipose tissue revealed an increase in *Cpt1a* and *Ucp2* and a decrease in *Hsl* and *Lpl* expression in the 5w HFD‐WPI‐fed mice, relative to HFD‐CAS‐fed mice (Figure 3c). Again, in older age, there were no differences detected between HFD‐CAS and HFD‐WPI‐fed mice (Figure 3g). No significant differences were detected in *Ctp1b* and *Ucp3* expression (Figures 3g and S2d). Conversely, the anabolic gene *Acc1* was expressed more in the HFD‐WPI group compared to the HFD‐CAS group during 10w, without any changes during 5w (Figure 3d,h). Only *Fatp1* was more highly expressed in both HFD adolescent groups, compared to the HFD adult groups (Figure S2e). The expression of the anabolic gene *Fasn* and the transporter *Cd36* remained unchanged.

Altogether, the results showed the specific effect of whey proteins within a HFD on leptin expression/plasma availability and on the expression of *Cd68*, *Fiaf* and some important genes involved in the catabolism of fatty acids in eWAT. However, these effects were only observed when the animals started the dietary intervention at 5‐week‐old; the same diet given at 10‐week‐old did not have the same effects.

### 3.5 Younger mice fed with HFD‐WPI have a higher proportion of *Lactobacillus murinus* within the gut

**Figure 4:**
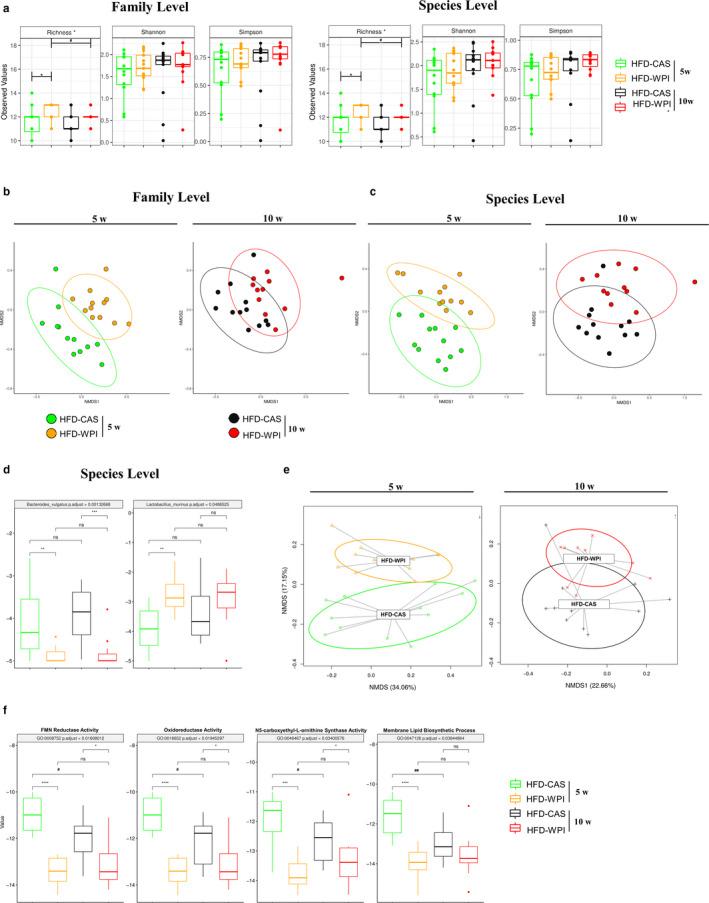
Age‐ and duration‐dependent effect of HFD‐WPI on the gut microbiota taxonomy and function. Taxonomic (a) alpha‐diversity at family and species level, measured with richness, Shannon and Simpson indexes, and beta‐diversity, calculated using NMSD ordination, both (b) at family and (c) species level, of HFD‐CAS and HFD‐WPI‐fed mice at both ages (5w and 10w). (d) Bar chart representing taxonomic differences at species level across the groups, using Kruskal–Wallis method. (e) Functional beta‐diversity calculated using NMSD ordination, and (f) bar chart representing differences in metabolic activities or processes across the HFD‐CAS and HFD‐WPI‐fed mice at both ages. *Statistical analysis*: groups showing * (for HFD‐CAS vs. HFD‐WPI) and # (5w vs. 10w) are significant (*/#*p* < .05 or **/##*p* < .01 or ***/###*p* < .001). A complete statistical description is detailed in Methods and Materials

Faecal DNA was subjected to shotgun metagenomic sequencing. The total number of reads per sample averaged 5,733,118 ± 1,768,632. These reads were used to investigate taxonomical and functional differences across the HFD‐fed mice at different ages. At the family and species level, the taxonomical analysis of alpha‐diversity indexes (i.e., Simpson and Shannon) did not differ across the groups, although Richness index was higher in 5w HFD‐WPI‐fed mice compared to their CAS counterpart with no differences between the two 10w groups (Figure 4a). The NMDS graph based on the dissimilarity matrix of Bray–Curtis showed clustering related to the type of diet. A clear separation between CAS and WPI groups was observed (Figures 4b,c, and S3b). At the family level, no significant differences were detected between HFD‐CAS and HFD‐WPI groups, either in 5w or in 10w (Figure 4b), even if the two younger groups showed a more evident separation (R^2^ = 0.14, *p* = .066 PERMANOVA pairwise analysis) compared to the older individuals. However, at species level, a significant separation was noted between 5w HFD‐CAS and HFD‐WPI (R^2^ = 0.147, *p* = .036; PERMANOVA pairwise analysis), with no differences between the two 10w counterparts (Figure 4c).

A significantly higher proportion of the family *Streptococcaceae* and the corresponding genus and species *Lactococcus* and *Lactococcus lactis* were present in all groups fed with a diet containing CAS as the main protein source (Figure S3a and S3c). This is in accordance with our previous study, in which we demonstrated that the high abundance of this taxa in mice fed with CAS is due to the presence of *L. lactis* in the diet (Boscaini et al., 2019). *Bacteroides vulgatus* was also more abundant in both CAS groups (Figures 4d and S3a). Interestingly, *Lactobacillus murinus*, was present in higher proportion in HFD‐WPI fed mice compared to their CAS counterparts during 5w, with no difference in the abundance of this species between the 10w groups (Figures 4d and S3a).

In addition to the taxonomical data, functional analyses were carried out. Alpha‐diversity analysis performed with HUMAnN2 did not show changes across the groups (data not shown). As for the taxonomic data, a clear separation between CAS and WPI functions in the NMDS graph based on the dissimilarity matrix of Bray–Curtis beta‐diversity plot was observed (Figures 4e and S3d). HFD‐WPI groups, at both ages, were significantly different compared to their CAS counterparts (5w: HFD‐CAS vs. HFD‐WPI R^2^ = 0.145, *p* = .006; 10w: HFD‐CAS vs. HFD‐WPI *R* ^2^ = 0.118, *p* = .012; PERMANOVA pairwise analysis) (Figure 4e). In addition, both WPI groups showed decreased metabolic FMN reductase, oxidoreductase and N5‐carboxyethil‐L‐ornithine synthase activities (Figure 4f). In contrast the membrane lipid biosynthetic process was decreased in 5w HFD‐WPI‐fed mice, relative to HFD‐CAS‐fed mice, with no difference between the 10w mice (Figure 4f).

**Figure 5:**
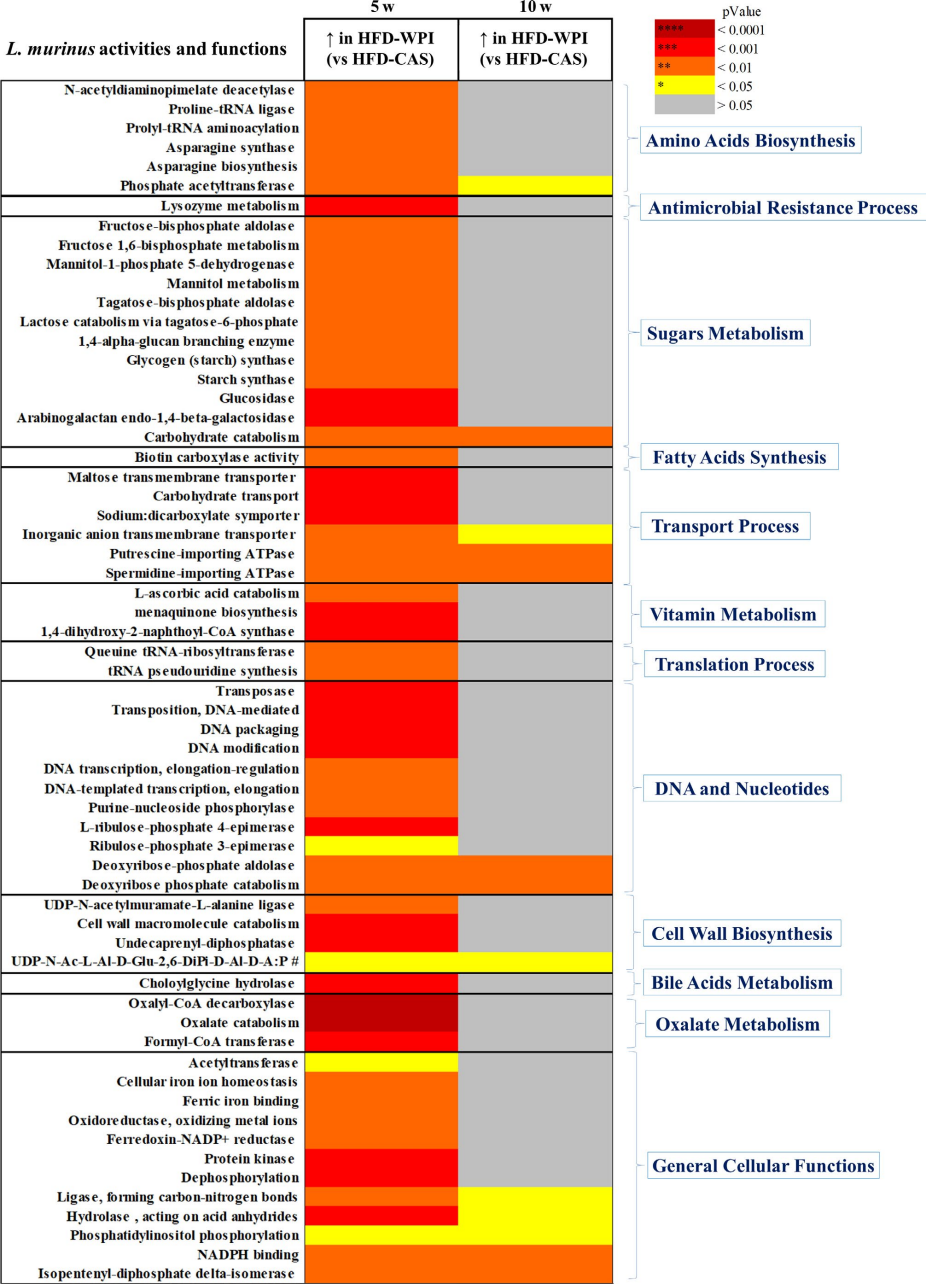
Differences in metabolic activities and processes of *Lactobacillus murinus*. Heat map showing significant differences in the increase of metabolic activities and processes abundance belonging to faecal *Lactobacillus murinus* in HFD‐WPI, relative to HFD‐CAS, in 5w compared to 10w mice. Abbreviation # (in cell wall biosynthesis): UDP‐N‐Ac‐L‐Al‐D‐Glu‐2,6‐DiPi‐D‐Al‐D‐A:P as UDP‐N‐acetylmuramoyl‐L‐alanyl‐D‐glutamyl‐meso‐2,6‐diaminopimelyl‐D‐alanyl‐D‐alanine:undecaprenyl‐phosphate transferase. *Statistical analysis*: groups showing * (for HFD‐CAS vs. HFD‐WPI) are significant (*p < .05, **p < .01, ***p < .001, ****p < .0001). A complete statistical description is detailed in Methods and Materials

In addition, we analysed in detail *L. murinus* ‐related functions that were significantly different across the groups. In total, we found 62 significantly different activities and functions across the groups (Figure 5) and, among these, 49 functions were significantly higher in HFD‐WPI‐fed mice compared to HFD‐CAS‐fed mice during 5w with no differences between the 10w groups. Several processes and functions, attributed to *L. murinus*, were found to be abundant within the younger HFD‐WPI group, such as amino acids biosynthesis, sugars metabolism (including glycolysis and fructose, mannose, lactose metabolism), vitamins metabolism (vitamin C catabolism and vitamin K2 biosynthesis), DNA and nucleic acid‐related processes (including pentose phosphate pathway) and oxalate metabolism (Figure 5). Altogether, these results suggest that the composition and the function of the intestinal bacterial population can be differently influenced based on the stage of life at which the dietary interventions start, together with other metabolic and physiologic outcomes.

All supplementary material available at https://figshare.com/s/4958026781f8a42c3f3e.

## 4 DISCUSSION

There is an increasing emphasis on understanding the microbiota function and composition in response to diet (Hughes, Marco, Hughes, Keim, & Kable, 2019). In particular, its modulation to a healthier configuration to ameliorate or prevent metabolic diseases, such as obesity, is of interest (Torres‐Fuentes, Schellekens, Dinan, & Cryan, 2015, 2017). Here, we have shown that a 5‐week‐diet intervention with HFD‐WPI, compared to HFD‐CAS, in mice that started the HFD intervention at 5‐weeks old, reduced weight gain and adiposity, changed eWAT catabolism and leptin production, with an increase in *Lactobacillus murinus* abundance and related functions within the gut. In contrast, none of these changes was observed in mice that underwent the same diet intervention starting from 10 weeks‐old and an extended period of WPI supplementation. These results suggest that the efficacy of this dietary intervention is age‐dependent and, based on age, the interplay with diet duration and the quality of the response is different. Thus, this is in accordance with our second hypothesis.

It has previously been shown that the presence of WPI as the protein component of a HFD caused a reduction in weight gain in rodents (McAllan et al., 2013, 2014; Pilvi et al., 2008). With the present paper, we confirmed a reduction in weight gain and adiposity in mice starting a 5‐week HFD‐WPI dietary intervention at 5‐week‐old. When the same dietary intervention was started at 10‐week‐old, the body weight trajectory and the cumulative weight gain of HFD‐WPI‐fed mice were not statistically significantly different, compared to their CAS counterparts. It is worth nothing though that while total weight gain was not significant, sWAT weight was significantly lower in HFD‐WPI compared to their CAS fed counterparts. Notably, WPI administration in LFD at 5‐week‐old did not prevent an increase in weight gain after the switch to HFD at 10‐week‐old. This finding goes against our first hypothesis.

In contrast with previous studies in rodents that showed a satiety effect due to WPI‐enriched diets as being the mechanism of action responsible for reduced weight gain (Hall, Millward, Long, & Morgan, 2003; Pezeshki, Fahim, & Chelikani, 2015; Zhou et al., 2011), here we found an increase in energy intake, both in HFD and LFD‐fed mice, suggesting a loss of energy during WPI supplementation. Notably, we also found a discrepancy between body weight and energy intake in our previous study, in which we were investigating the metabolic and gut microbial differences in mice fed with HFD containing alpha‐lactalbumin, a specific whey protein, compared to CAS counterparts (Boscaini et al., 2019). To investigate the loss of energy that occurred between WPI and CAS groups, we measured the level of TAG in the caecum. Variation of fat, specifically free fatty acids, cholesterol, and TAG, in faeces is an indication of intestinal function and fat absorption changes under certain conditions (Kraus, Yang, & Kahn, 2015; Mataki et al., 2007). No differences were detected in the TAG levels between WPI and CAS‐fed mice, but we cannot exclude that free fatty acids or cholesterol can be involved in the afore‐mentioned loss of energy.

Surprisingly, during organ weight data analysis, we observed that the liver was heavier in the presence of WPI within the diet. Since we did not observe either an accumulation of hepatic TAG or an overexpression of genes involved in fatty acids biosynthesis in the liver of WPI‐fed mice, we could not explain the higher weight of the liver in these mice. Perhaps, this phenomenon is due to inflammation or an accumulation of other macromolecule such as glycogen or cholesterol or vitamins (Bhattacharya, 2015; Li, Cordero, Nguyen, & Oben, 2016). Future investigation, using histological and transcriptomics approaches, will be required to fully elucidate the effect of WPI on liver weight.

In previous studies from our group it was observed that a LFD enriched in WPI changed the gene expression of some ileal nutrient transporters and satiety‐control hormones, such as FATP4, methionine transporter and PYY (McAllan et al., 2013; Nilaweera et al., 2017). In the present study, we showed that, with the exception of *Glut2* in 10w, WPI provided in presence of HFD decreases the capacity to regulate the expression of ileal nutrient transporters and satiety hormones. Instead, some of the analysed genes (i.e., *Cd36*, *Fatp4*, *Pyy*, *Gcg*) underwent increased expression in 5w relative to 10w mice. In accordance with these data, plasma levels of TAG, glucose and insulin also remained unchanged between WPI and CAS across the groups, but they showed some variations with age. This is in line with other studies testing TAG, glucose and insulin during HFD‐WPI administration (McAllan et al., 2013; Pilvi et al., 2007). The fact that nutrient transporter expression and plasma metabolites are different between mice at different ages might be because they started the HFD dietary intervention at different stages of life with different duration of WPI intake. Consequently, we suggest that the decreased weight gain and adiposity observed in the presence of WPI‐enriched HFD at younger age is not controlled or modulated by a differential nutrient absorption mechanism within the small intestine.

Our data suggest that decreases in weight gain and adiposity in younger mice fed a HFD‐WPI might be linked with eWAT catabolism. *Cpt1a* and *Ucp2* are genes coding, respectively, for a mitochondrial enzyme that catalyses the rate‐limiting step of fatty acids β‐oxidation and a mitochondrial membrane protein that dissipates metabolic energy with prevention of oxidative stress accumulation (Bäckhed, Manchester, Semenkovich, & Gordon, 2007; Horvath et al., 2003; Pierelli et al., 2017; Warfel et al., 2017). While these two genes were expressed more in mice fed a HFD‐WPI when younger, *Hsl* and *Lpl* genes, together with the gene coding for LPL‐fasting induced inhibitor FIAF (also involved in triglyceride metabolism), were expressed less in the same group, compared to 5w mice fed with HFD‐CAS. HSL is an intracellular neutral lipase responsible for the hydrolysis of TAG, diacylglycerols, monoacylglycerols, and cholesteryl‐esters (Haemmerle, 2006; Shen et al., 2011) and LPL is an enzyme that hydrolyses triglycerides (TG) and controls the kinetics and transport of the majority lipoproteins in the plasma (Wang & Eckel, 2009). This suggests that WPI modulates the expression of key genes involved in adiposity control and that play a role in metabolic syndrome development, thereby accelerating eWAT fatty acids catabolism and ameliorating adiposity and HFD‐induced obesity phenotype.

In 5w mice fed with HFD‐WPI, the level of expression of the eWAT *Ob* gene, together with the plasma level of the satiety hormone produced by this gene, leptin, were lower compared to the CAS counterparts. Notably, plasma leptin, but not *Ob* expression, was also lower in adult HFD‐WPI‐fed mice compared to the CAS controls. Also in the presence of LFD, WPI have been shown to reduce plasma levels of leptin (McAllan et al., 2014); however, we found no differences in circulating leptin between LFD‐WPI and LFD‐CAS, after 10 weeks of dietary intervention. As is well established for HFD‐induced obesity in mice and obese humans, leptin signalling to the brain is disrupted and, consequently, its level in periphery is higher than in the healthy status (Zhou & Rui, 2013). WPI, not only by influencing fatty acids catabolism, but also possibly by lowering both plasma leptin and *Ob* gene expression, positively modulate adiposity in mice and this effect seems to be more powerful in the presence of HFD than LFD. Notably, our data suggest that a reduction in plasma leptin in HFD‐WPI‐fed mice is directly due to a reduction in *Ob* gene expression, rather than a reduction in adipose mass. Low *Cd68* expression in eWAT, an inflammatory marker, gives a first insight regarding a low inflammatory state within the adipose tissue in the presence of HFD‐WPI, which is usually very high in mice and humans with obesity (Stolarczyk, 2017).

Aside from eWAT catabolism and leptin production, the gut microbiota composition and function showed changes between 5w HFD‐WPI‐ and HFD‐CAS‐fed mice, that were no longer observed between 10w HFD groups. Specifically, the most important difference lay at species‐level, where the species *Lactobacillus murinus* showed an increased abundance in younger HFD‐WPI‐fed mice relative to the CAS controls. *L. murinus* can attenuate allergic responses in mice with food allergy‐induced dysbiosis within the gut microbiota and its high proportions in the gut are correlated with a lower degree of necrotizing enterocolitis, suggesting a protective role in the intestine (Huang, Shen, Liang, & Jan, 2016; Isani et al., 2018). In addition, a recent study showed that there was a high proportion of *L. murinus* in the gut of mice that underwent calorie restriction, which was also linked with anti‐inflammatory effects. In the same study, it was also demonstrated that *L. murinus* reduced intestinal permeability and systemic inflammatory markers in old microbiota‐colonized gnotobiotic mice (Pan et al., 2018).

In this study, *L. murinus* levels within the gut of younger HFD‐WPI‐fed mice were linked with changes in the functional profile, such as upregulation of amino acids, fatty acids, and cell wall biosynthesis functions, together with functions related to transport, translation, DNA, nucleotides, sugar metabolism and general cellular function. This can be explained by the fact that WPI stimulates the growth of gut *L. murinus*, thus all the bacterial functions related to growth, cell division and survival are upregulated. Additionally, oxalate, bile acid, and vitamin metabolism were found to be *L. murinus*‐related upregulated functions in the presence of WPI.

Oxalate is a toxic compound found in fruit, vegetables, grains, and nuts. Excessive concentration of oxalate can cause problems in the gastrointestinal tract and exceptionally kidney failure. Recently, probiotic bacteria belonging to *Bifidobacterium* and *Lactobacillus* have been studied for their potential to degrade oxalate. A direct correlation between the oral administration of probiotic bacteria and the reduction of urinary oxalate excretion has been observed in rats and humans (Noonan & Savage, 1999; Turroni et al., 2007).

Gut bacterial vitamin and bile acids metabolism are among the most important examples of gut bacteria‐host metabolism interaction. In particular, it was recently shown that HFD causes a serious alteration of the bile acid pool, that may contribute to obesity morbidity and development (Lin, An, Tang, & Wang, 2019).

This work builds upon a decade of our research where we have tried to understand the energy balance related effects of WPI, which have led to the finding that these proteins can reduce adiposity and body weight without relying on energy intake. Here, we have shown that early nutritional intervention with whey proteins alter the composition of the gut microbiota and adiposity, where the effects of the catabolic response in the tissue were evident, suggesting a potential functional relationship between changes in the gut and the adipose tissue, which is not seen in later stage of the mouse life despite prolong intake of the proteins. It is interesting that preterm babies fed whey proteins gain weight but this effect is lost in term babies (Berger, Scott, Kenward, Scott, & Wharton, 1979). More recently, it has been shown that babies aged 28 day fed formula with a higher proportion of whey proteins consume more of the formula but gain less weight (gain per intake) than babies fed low amounts of the proteins (Fleddermann et al., 2014).

Taken together our findings show that WPI exerts a possible prebiotic effect within the gut thanks to its ability to promote *L. murinus* particularly at a young age. Consequently, we propose that the role of WPI, during HFD administration, in the amelioration of weight gain and regulation of adipose tissue metabolism might be directly linked to modulation of the gut microbiota. Future work is planned, which aims to address this hypothesis.

## CONFLICT OF INTERESTS

The authors declare no conflict of interest.

## AUTHOR CONTRIBUTIONS

K. N. N., P. D. C., J. R. S., J. F. C. and S.B. designed the study. S. B. and K.N.N performed the animal experiment. S.B. performed all the experiments. O.N. helped to perform some experiments and statistical analyses. R. C. R. carried out the bioinformatics analyses and related statistics. S.B. and R.C.R generated the figures. All authors contributed to the drafting of the manuscript. All the authors approved the final version for submission.

## Supporting information



Fig S1

Fig S2

Fig S3

Fig S4

Fig S5
