# Supplementary figures and images for: Age‐ and duration‐dependent effects of whey protein on high‐fat diet‐induced changes in body weight, lipid metabolism, and gut microbiota in mice
Source: Physiol Rep. 2020 Aug 3;8(15):e14523. doi: 10.14814/phy2.14523 (PMC7399378; doi:10.14814/phy2.14523)

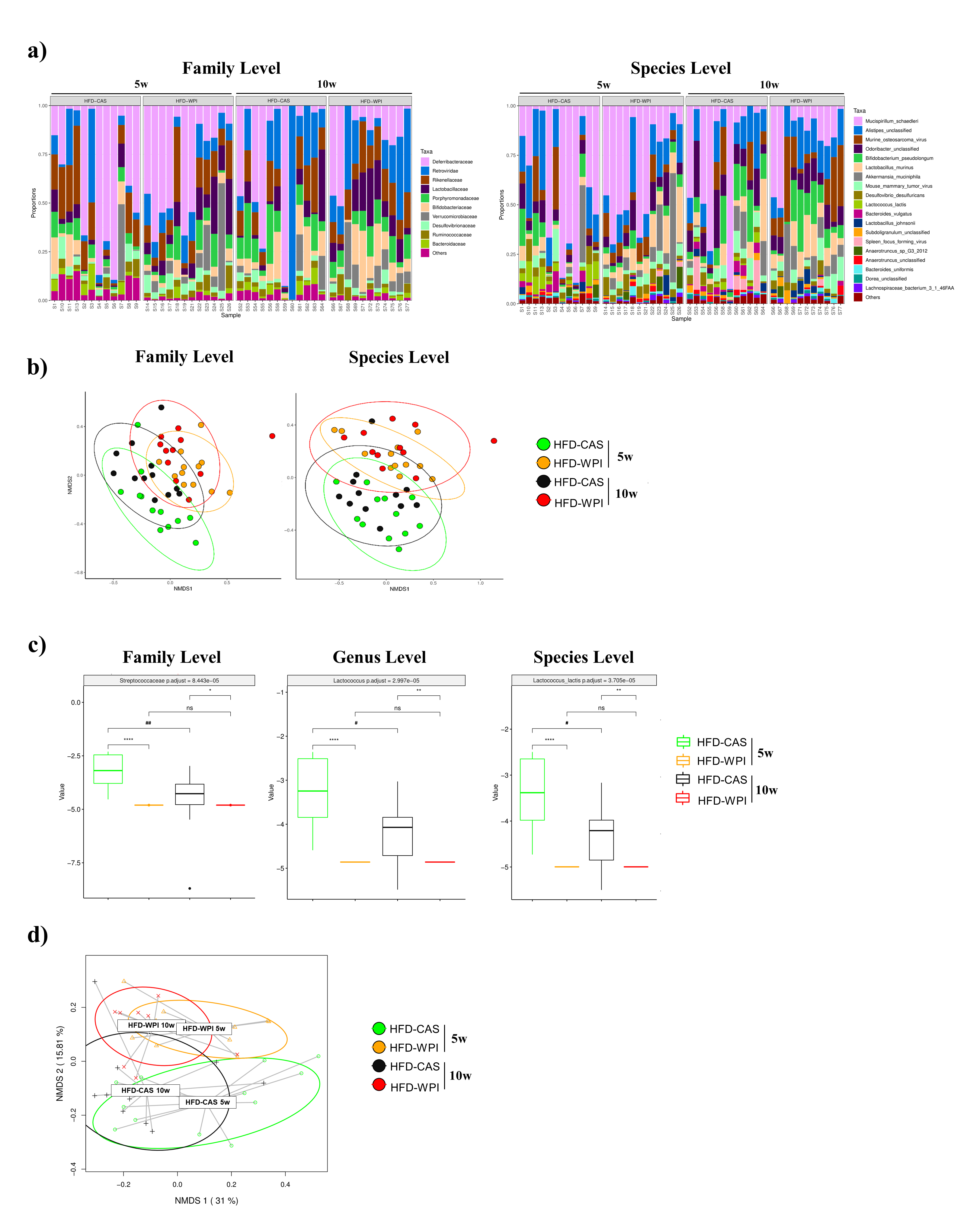

Supplement: Supplementary file 3 — Fig S3 [file PHY2-8-e14523-s003.tif]

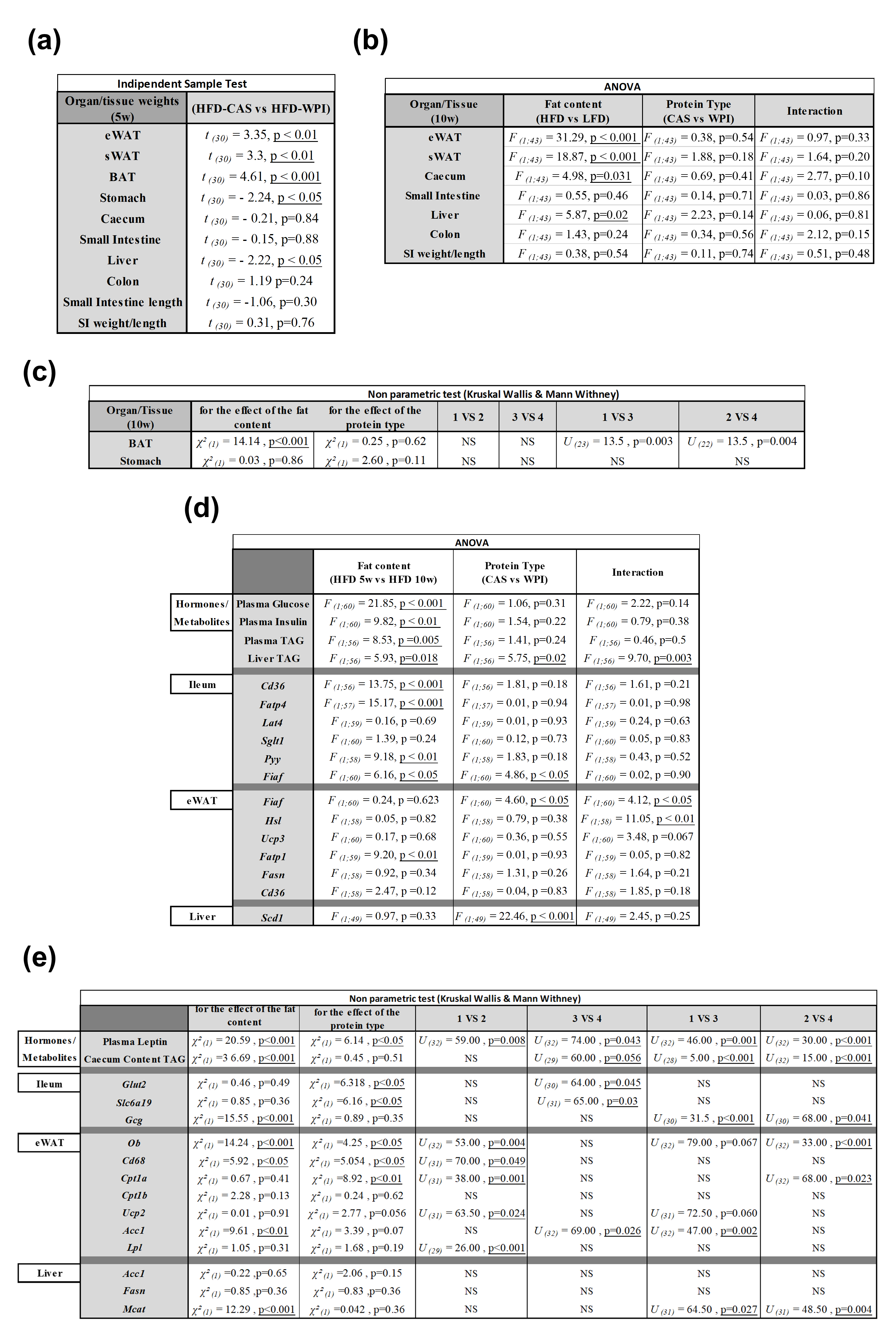

Supplement: Supplementary file 4 — Fig S4 [file PHY2-8-e14523-s004.tif]

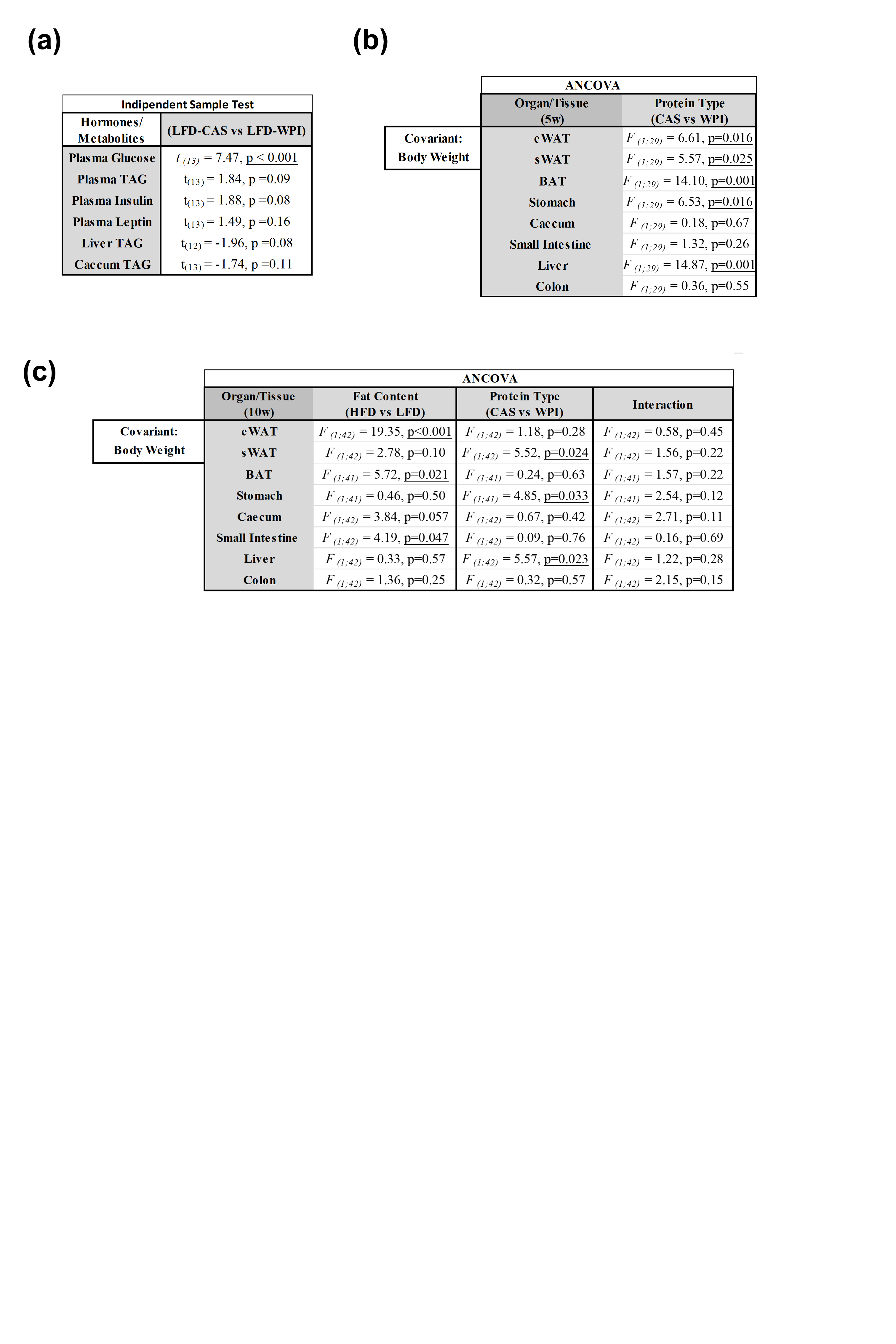

Supplement: Supplementary file 5 — Fig S5 [file PHY2-8-e14523-s005.tif]
