# Supplementary material for: Age‐ and duration‐dependent effects of whey protein on high‐fat diet‐induced changes in body weight, lipid metabolism, and gut microbiota in mice
Source: Physiol Rep. 2020 Aug 3;8(15):e14523. doi: 10.14814/phy2.14523 (PMC7399378; doi:10.14814/phy2.14523)
Supplement: Supplementary file 6 [file PHY2-8-e14523-s006.docx]

**Supplemental Figure Legends**

**Figure S1: Plasma metabolites and hormones, and TAG in the caecum and in the liver.** Data show triacylglycerol (TAG) level in the caecum **(a)** between HFD groups in 5w vs 10w and **(b)** between LFD-CAS and LFD-WPI groups. TAG level in the liver **(c)** between HFD groups in 5w vs 10w and **(d)** between LFD-CAS and LFD-WPI groups. Plasma levels of TAG **(e)** between HFD groups in 5w vs 10w and **(f)** between LFD-CAS and LFD-WPI groups. Plasma levels of glucose **(g)** between HFD groups in 5w vs 10w and **(h)** between LFD-CAS and LFD-WPI groups. Plasma levels of insulin **(i)** between HFD groups in 5w vs 10w and **(j)** between LFD-CAS and LFD-WPI groups. Plasma levels of leptin **(k)** between HFD groups in 5w vs 10w and **(l)** between LFD-CAS and LFD-WPI groups. *Statistical analysis*: groups showing * (for HFD-CAS vs HFD-WPI) and # (HFD-CAS 5w vs HFD-CAS 10w and HFD-WPI 5w vs HFD-WPI 10w) are significant (*/# P<0.05 or **/## P<0.01 or ***/### P<0.001). A complete statistical description is detailed in Methods and Materials and in figures S4 and S5.

**Figure S2: Hepatic, Ileal and eWAT gene expression.** Data show **(a)** expression of genes coding for enzymes involved in fatty acids biosynthesis within the liver, **(b)** ileal gene expression of hormones and nutrient transporters, **(c)** expression of genes coding for leptin, inflammation marker CD68 and the FIAF, **(d)** catabolic and **(e)** anabolic enzymes in the eWAT. Abbreviations: ACC1; acetyl-CoA carboxylase 1, FASN; fatty acids synthase; MCAT, malonyl CoA-acyl carrier protein transacylase; SCD1, stearyl-CoA desaturase; other figure descriptions in figures 2 and 3. *Statistical analysis*: groups showing * (for HFD-CAS vs HFD-WPI) and # (HFD-CAS 5w vs HFD-CAS 10w and HFD-WPI 5w vs HFD-WPI 10w). A complete statistical description is detailed in Methods and Materials and in figures S4.

**Figure S3:** **Effect of HFD-WPI in the gut microbiota of 5w vs 10w** **mice.**

**(a)** Taxaplot showing families and species within the faecal samples of 5w and 10w mice fed with HFD-CAS and HFD-WPI. Also reported **(b)** taxonomic beta-diversity, calculated using NMSD ordination, both at family (R^2^=0.146, pValue=0.001) and species level (R^2^=0.181, pValue=0.001), **(c)** bar charts representing taxonomic differences at family, genus and species level across the HFD groups (5w and 10w), using Kruskal Wallis method. The last graph shows **(d)** functional beta-diversity (R^2^=0.174, pValue=0.001) calculated using NMSD ordination, across the HFD groups (5w and 10w). *Statistical analysis*: groups showing * (for HFD-CAS vs HFD-WPI) and # (HFD-CAS 5w vs HFD-CAS 10w and HFD-WPI 5w vs HFD-WPI 10w) are significant (*/# P<0.05 or **/## P<0.01 or ***/### P<0.001 or ****/#### p<0.0001). A complete statistical description is detailed in Methods and Materials.

**Figure S4: Statistical analysis details.** Complete statistical description organs and tissues weight data **(a)** in 5w and **(b)(c)** in 10w. Complete statistical description of **(d)(e)** hormones and metabolites data, and gene expression data. Mann Withney *U* test: 1= HFD-CAS 5w; 2= HFD-WPI 5w; 3 = HFD-CAS 10w, 4 = HFD-WPI 10w.

**Figure S5:** **Hormones and metabolites statistics, and tissue/organs weight ANCOVA.** Complete statistical description of **(a)** hormones and metabolites data in LFD-fed mice and organs and tissues weight ANCOVA **(b)** in 5w and **(c)** in 10w.

**Table ST1:** Sequences of qPCR primers.
