# Supplementary material for: Age‐ and duration‐dependent effects of whey protein on high‐fat diet‐induced changes in body weight, lipid metabolism, and gut microbiota in mice
Source: Physiol Rep. 2020 Aug 3;8(15):e14523. doi: 10.14814/phy2.14523 (PMC7399378; doi:10.14814/phy2.14523)
Supplement: Supplementary file 7 [file PHY2-8-e14523-s007.docx]

**Supplementary Statistic**

The outliers were calculated and excluded performing Grubbs’ test (significance level = 0.05).

**Repeated Measurement**

Body weight in experiment 5w (0-5 weeks): *F_(5;215)_* = 436.71, p < 0.001 for the effect of the time, *F_(1;43)_* = 6.14, p < 0.05 for the effect of the diet fat content, *F_(1;43)_* = 2.38, p > 0.05 for the effect of the protein type, *F_(5;215)_* = 21.43 , p < 0.001 for the effect of the time x diet fat content interaction, *F_(5;215)_* = 2.54, p > 0.05 for the effect of the time x diet protein type interaction, *F_(5;215)_* = 3.12, p < 0.05 for the effect of the time x diet fat content x diet protein type interaction.

Body weight in experiment 10w (0-10 weeks): *F_(10;430)_* = 339.36, p < 0.001 for the effect of the time, *F_(1;43)_* = 3.21, p > 0.05 for the effect of the diet fat content, *F_(1;43)_* = 0.15, p > 0.05 for the effect of the protein type, *F_(10;430)_* = 30.83 , p < 0.001 for the effect of the time x diet fat content interaction, *F_(10;430)_* = 0.53, p > 0.05 for the effect of the time x diet protein type interaction, *F_(10;430)_* = 3.11, 0.07 < p > 0.05 for the effect of the time x diet fat content x diet protein type interaction.

Cumulative Weight Gain experiment 5w (0-5 weeks): *F_(_**_4;172)_* = 340.66, p < 0.001 for the effect of the time, *F_(1;43)_* = 33.29, p < 0.001 for the effect of the diet fat content, *F_(1;43)_* = 4.13, p < 0.05 for the effect of the protein type, *F_(4;172)_* = 12.55 , p < 0.001 for the effect of the time x diet fat content interaction, *F_(4;172)_* = 1.36, p > 0.05 for the effect of the time x diet protein type interaction, *F_(4;172)_* = 2.55 p > 0.05 for the effect of the time x diet fat content x diet protein type interaction.

Cumulative Weight Gain experiment 10w (0-10 weeks): *F_(_**_9;396)_* = 285.36, p < 0.001 for the effect of the time, *F_(1;44)_* = 16.91, p < 0.001 for the effect of the diet fat content, *F_(1;44)_* = 0.026, p > 0.05 for the effect of the protein type, *F_(9;396)_* = 37.85, p < 0.001 for the effect of the time x diet fat content interaction, *F_(9;396)_* = 0.66, p > 0.05 for the effect of the time x diet protein type interaction, *F_(9;396)_* = 4.61 p < 0.05 for the effect of the time x diet fat content x diet protein type interaction.

Cumulative Energy Intake/mouse experiment 5w (0-5 weeks): *F_(_**_9;72)_* = 6615.09, p < 0.001 for the effect of the time, *F_(1;8)_* = 8.09, p < 0.05 for the effect of the protein type,  *F_(9;72)_* = 20.71, p < 0.01 for the effect of the time x diet protein type interaction.

Cumulative Energy Intake/mouse experiment 10w (0-10 weeks): *F_(4;32)_* = 3575.00, p < 0.001 for the effect of the time, *F_(1;8)_* = 11.36, p < 0.05 for the effect of the protein type, *F_(4;32)_* = 8.70, p < 0.05 for the effect of the time x diet protein type interaction.
